# Impact of the COVID-19 Pandemic on the Effectiveness of a Metabolic Health Telemedicine Intervention for Weight Loss: A Propensity Score Matching Analysis

**DOI:** 10.3389/fpubh.2022.897099

**Published:** 2022-06-16

**Authors:** Shaminie J. Athinarayanan, Rebecca N. Adams, Michelle VanTieghem, Amy L. McKenzie, Brittanie M. Volk, Robert E. Ratner, Stephen D. Phinney

**Affiliations:** Virta Health, San Francisco, CA, United States

**Keywords:** weight loss, COVID-19, pandemic, nutritional ketosis, telemedicine

## Abstract

**Background:**

Coronavirus disease 2019 (COVID-19) pandemic public health measures such as stay-at-home and mandatory work-from-home orders have been associated with obesogenic lifestyle changes, increased risk of weight gain, and their metabolic sequelae. We sought to assess the impact of this pandemic on weight loss from a telemedicine-delivered very-low-carbohydrate intervention targeting nutritional ketosis (NKI).

**Methods:**

A total of 746 patients with a BMI ≥25kg/m^2^, enrolled between January and March 2020 and treated for at least 1 year with the NKI, were classified as pandemic cohort (PC). A separate cohort of 699 patients who received 1 year of the NKI in the preceding years, enrolled between January and March 2018, were identified as pre-pandemic cohort (Pre-PC). Demographic and clinical data were obtained from medical records to compare the cohorts and assess the outcomes. Using propensity score matching (PSM), balanced and matched groups of 407 patients in the Pre-PC and 407 patients in the PC were generated. Longitudinal change in absolute weight and percentage weight change from baseline to 1 year were assessed.

**Results:**

Weight significantly decreased in both PC and Pre-PC at 3, 6, 9, and 12 months. The weight loss trajectory was similar in both PC and Pre-PC with no significant weight differences between the two cohorts at 3, 6, 9, and 12 months. On an average, the PC lost 7.5% body weight while the Pre-PC lost 7.9% over 1 year, and the percent weight loss did not differ between the two cohorts (*p* = 0.50).

**Conclusion:**

A very-low-carbohydrate telemedicine intervention delivered comparable and medically significant weight loss independent of pandemic stress and lifestyle limitations.

## Introduction

The declaration of Coronavirus disease 2019 (COVID-19) as a pandemic on 11 March 2020 resulted in the implementation of public health measures such as stay-at-home orders, working-from-home mandates, and social distancing. Though important in controlling the virus spread, these measures contributed to obesogenic changes in lifestyle and daily routines such as increased food consumption, frequent snacking, reduced physical activity, and decreased sleep quality (1–3). An overall trend for weight gain has been reported in the general US population at 1.5 pounds per month (4). These negative lifestyle changes are particularly deleterious in individuals with pre-obesity or obesity, who are highly susceptible to severe COVID-19 infection with worse outcomes related to mortality and hospitalization (5, 6). Moreover, individuals with obesity are more vulnerable to the negative effects of social isolation or quarantine (7, 8). The radical change in lifestyle during the pandemic not only impacted obesity management (39, 10) but also increased the risk for weight regain in already highly susceptible individuals (9–11).

Frequency of on-site face-to-face obesity management services (12, 13) and bariatric surgeries were reduced or canceled during the lockdown period (13, 14). This further impacted individuals with obesity who were actively seeking treatment for weight loss, those who were already enrolled in a weight loss program, and patients in post-bariatric operative care. Consequently, to address this issue, weight loss treatment delivered *via* telemedicine or through online classes became more common (15–17). These digitally delivered weight loss programs were effective in eliciting short-term weight loss during the pandemic (15, 16). Furthermore, the pandemic also transformed the provision of medical care, especially in the management of various chronic diseases, by promoting the use and implementation of telemedicine (18–20).

So far, no studies have evaluated the effect of the pandemic on weight loss over 1 year. Virta Health is a US-based telemedicine clinic that provides care for adults with type 2 diabetes (T2D), prediabetes, and obesity using a medically supervised very-low-carbohydrate intervention with nutritional ketosis (NKI). Patients are treated and monitored using a continuous remote telemedicine platform. Patients have access to a mobile health application (app) through which they have access to educational materials, track biomarkers, communicate with their medical care team consisting of health coaches and licensed medical providers, and have the option of interacting with peers *via* a social community.

In this study, using retrospective data from Virta Health medical records, we explored whether patients receiving a very-low-carbohydrate telemedicine intervention lost the same amount of weight during the pandemic as an independent cohort who received the same telemedicine treatment in the period prior to the pandemic. To address baseline imbalance between the two retrospective cohorts, we used propensity score matching (PSM) to generate matched cohorts for the final analysis.

## Methods

### Study Details

This is a retrospective cohort study assessing the effect of the pandemic on the efficacy of a telemedicine intervention on weight loss. In this study, patients treated at Virta Health were assessed using de-identified demographic, clinical, and laboratory data obtained from medical records. Weight was tracked with a cellular connected scale that automatically uploaded to the app (Body Trace BT003 New York, USA), and patients were encouraged to regularly upload fingerstick blood glucose and beta-hydroxybutyrate (BHB) results to monitor their progress in the treatment and adherence to NKI, respectively. Two independent cohorts of patients with BMI ≥25kg/m^2^ were retrospectively identified from the database. Pandemic cohort (PC) patients enrolled in the NKI treatment between January and March 2020 and were treated for at least 1 year, while pre-pandemic cohort (Pre-PC) patients received the NKI treatment for at least a year and enrolled between January and March 2018. To assess the effect of the pandemic independently on weight loss, the enrollment and treatment period of these two cohorts were non-overlapping and seasonality effects were controlled for by choosing the same enrollment quarter.

### Outcome Measures

De-identified baseline demographics and clinical and daily app data, including gender, age, baseline weight, baseline glycated Hemoglobin A1c (HbA1c), weight data, and other app-usage variables (total days with BHB logs, total days with weight logs, and total days of app usage from baseline to 1 year), were obtained for this analysis. Weight at 3–, 6–, 9–, and 12–month milestones represents the weight closest to the time point within a 90-day window (3 months; 60–149 days, 6 months; 150–239 days, 9 months; 240–330 days and 12 months; 335–425 days). Reported diagnosis of and hospitalization for COVID-19 were also included.

The primary outcome was the difference in weight change from baseline to 1 year between the Pre-PC and PC. Secondary outcomes included difference in percentage weight change at 1 year from baseline, including the proportion of patients who achieved clinically meaningful weight loss, and difference in app usage between the Pre-PC and PC. Percentage weight change was defined as weight change at 1 year (weight at 1 year minus baseline weight) divided by baseline weight and multiplied by 100.

### Statistical Analysis

To reduce bias and imbalance in the unmatched Pre-PC and PC dataset, we used PSM to create matched PC and Pre-PC. Propensity scores were generated by aggregating multiple baseline demographics and clinical variables into a single dimension. The propensity score was calculated using logistic regression, and the predicted probability of participants to be assigned in the Pre-PC and PC were estimated using selected baseline characteristics. Baseline variables included in the PSM analysis were age, gender, diagnosis, baseline body mass index (BMI), and baseline HbA1c. Nearest-neighbor matching with a 1:1 ratio using a fixed caliper width (0.02) was used to create a matched Pre-PC and PC dataset. To assess balance of covariate distribution between the propensity score matched Pre-PC and PC, standardized differences were used as a diagnostic variable. An absolute value of standardized difference greater than 0.1 was considered a sign of imbalance. Independent sample *t*-tests and Chi-square tests were used to assess the differences in the baseline characteristics of continuous and categorical variables between the unmatched and matched Pre-PC and PC. Continuous variables were expressed as Mean (SD) or Mean ± SE, and categorical variables were listed as percentage.

The primary outcome comparison between matched Pre-PC and PC was performed using a linear mixed effects model (LMM) for absolute weight change from baseline to 1 year (including weight at baseline, 3, 6, 9, and 12 months). An independent samples *t*-test was used to assess difference in percent weight change at 1 year between matched Pre-PC and PC. Tests for differences in percent weight change were performed with missing weight values imputed and among only those with weight data recorded within the specified time period. As an exploratory analysis, we used Chi-square tests to compare the proportions of patients achieving clinically significant weight loss of 5% or more and 10% or more in Pre-PC and PC, respectively. Finally, we evaluated the differences in the app-use between Pre-PC and PC with independent *t*-tests. Statistical significance was defined as *p* < 0.05. Statistical analyses were performed using SPSS software version 28 (SPSS 28.0 IBM Corporation, Armonk, New York, USA), and the PSM analysis in SPSS was performed using the R plugin.

## Results

### Baseline Characteristics and PSM

**Figure 1:**
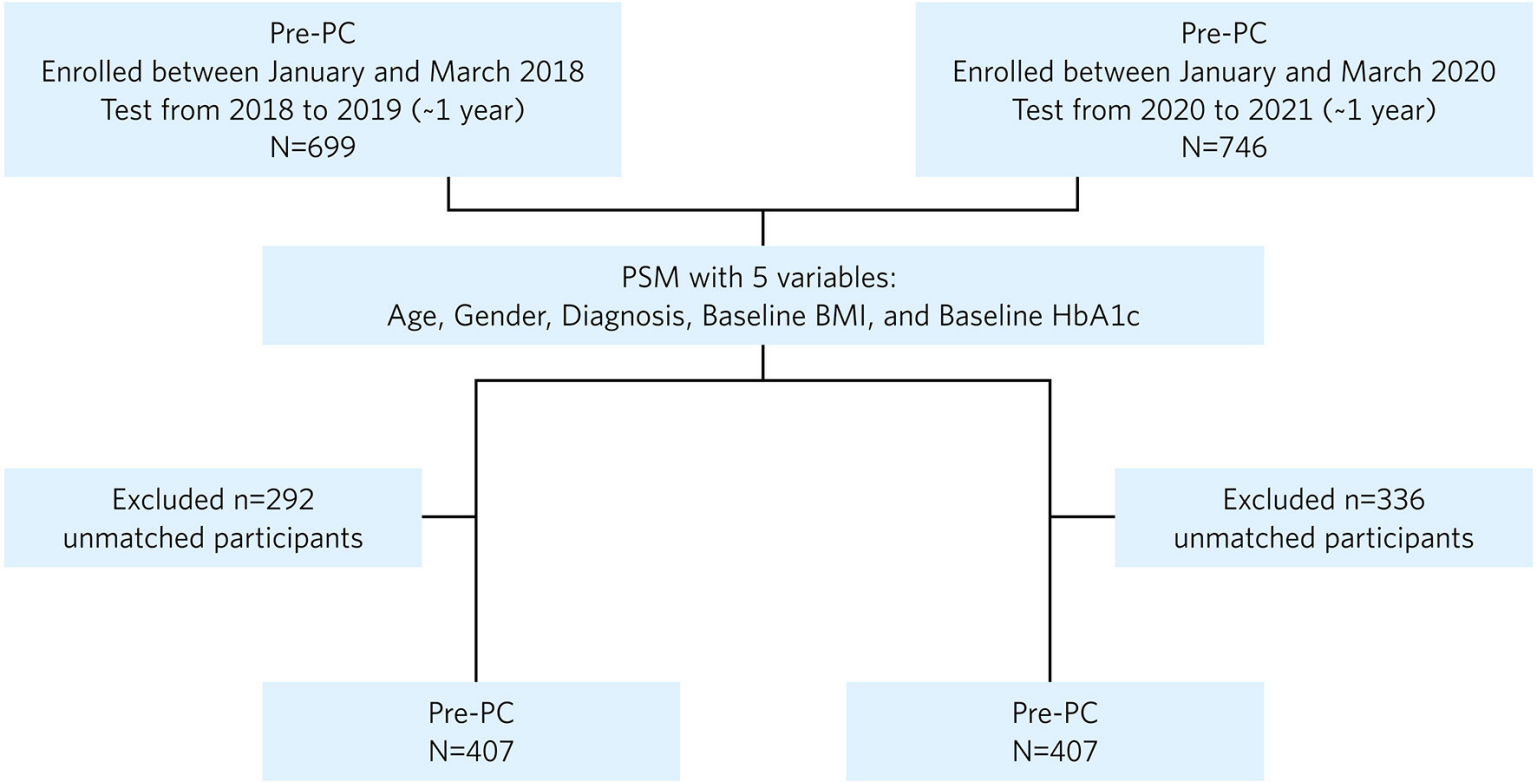
Flowchart of patients included in the final analysis after propensity score matching (PSM). PC, pandemic cohort; Pre-PC, pre-pandemic cohort; PSM, propensity score matching.

This study included 699 Pre-PC and 746 PC participants (Figure 1). The baseline characteristics of the unmatched Pre-PC and PC participants are described in Table 1. Baseline assessment showed imbalance between the unmatched Pre-PC and PC, where the PC had a significantly greater proportion of males and individuals with T2D, higher baseline HbA1c, and lower baseline BMI than the Pre-PC. The PSM analysis resulted in 407 Pre-PC and 407 PC matched participants, who were included in the final analyses (Figure 1). No statistically significant differences were observed in any of the baseline demographics and clinical variables between the matched Pre-PC and PC (Table 1). Matching using propensity scores also generated a balanced Pre-PC and PC cohorts as assessed by absolute standardized differences, where none of the means and prevalence of baseline covariates were greater than 0.10 (Table 1). Within the PC, 64 (15.7%) participants reported a COVID-19 diagnosis within the 1-year intervention period, and 5 of the 64 (8%) required hospitalization.

**Table 1:** Baseline characteristics of unmatched and matched pre-pandemic cohort (Pre-PC) and pandemic cohort (PC).

**Baseline characteristics**	**Unmatched cohort**, ***n*** **=** **1,445**			**Propensity matched cohort**, ***n*** **=** **814**			
	**Pre-PC, *n* = 699**	**PC, *n* = 746**	***P*-value**	**Pre-PC, *n* = 407**	**PC, *n* = 407**	***P*-value**	**Standardized differences**
Age (years)	53.2 (9.1)	53.6 (8.4)	0.33	53.8 (8.6)	53.7 (8.4)	0.85	0.01
Female (%)	52.9	43.3	**<0.001**	45.9	47.2	0.73	−0.03
**Enrollment diagnosis**							
T2D, *n* (%)	418 (59.8)	697 (93.4)	**<0.001**	366 (89.9)	366 (89.9)	1.00	0.00
Prediabetes, *n* (%)	275 (39.3)	45 (6.0)	**<0.001**	41 (10.1)	41 (10.1)	1.00	0.00
Other, *n* (%)	6(0.9)	4 (0.5)	NA	NA	NA	NA	NA
BMI (kg/m^2^)	37.2 (7.2)	36.0 (7.6)	**<0.001**	36.2 (6.8)	36.3 (7.7)	0.92	−0.01
Weight (kg)	109.6 ± 0.9	107.8 ± 0.9	0.17	108.0 ± 1.2	108.7 ± 1.2	0.65	−0.03
HbA1c (%)	6.9 ± 0.1	7.9 ± 0.1	**<0.001**	7.5 ± 0.1	7.5 ± 0.1	0.85	0.00
COVID-19 diagnosis (%)				NA	15.7		

*Pre-PC, Pre-pandemic cohort; PC, Pandemic cohort; T2D, type 2 diabetes; BMI, body mass index; HbA1c, hemoglobin A1c; NA, not available. Independent sample T-tests were used to compare means between Pre-PC and PC. All patients included in the analysis had a BMI ≥ 25kg/m^2^ with either pre-obese or obese. The diagnosis categories listed in Table 1 were enrollment diagnoses listed in their medical chart when the patient registered and enrolled in Virta health treatment. Patients who did not have T2D or prediabetes at enrollment were listed under other category. Statistical comparison of the proportion with uncomplicated pre-obesity or obesity within pre-PC and PC was not performed given the limited number of individuals. An absolute standardized difference of >0.10 was considered different and the variable as imbalanced. The bold values indicates the values of p < 0.001 are statistically significant*.

### Weight and Percentage Weight Loss Outcomes

**Figure 2:**
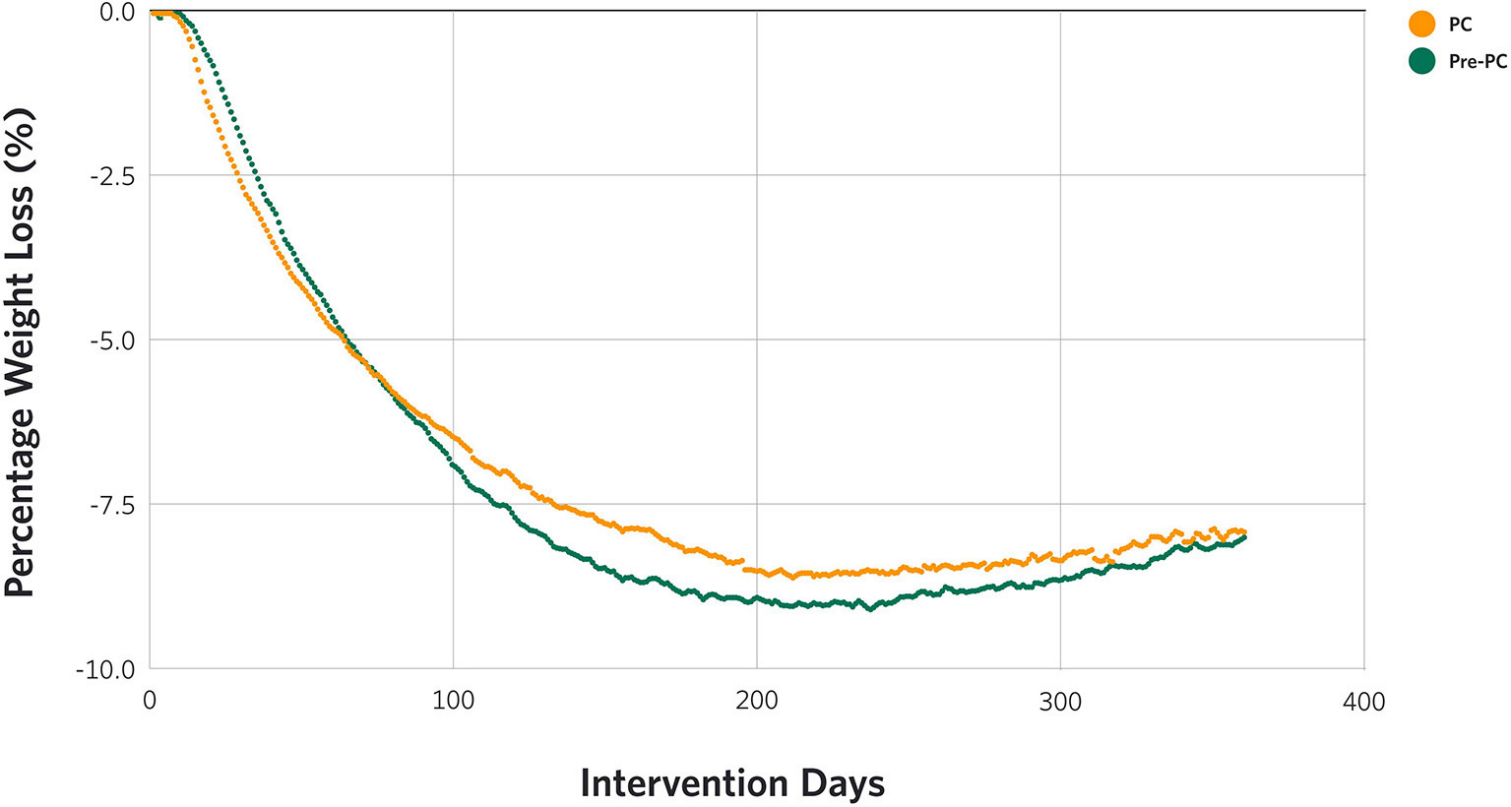
Percentage weight loss (%) trend during 1 year of the intervention in Pre-PC and PC.

There were statistically significant reductions in weight from baseline to 3, 6, 9, and 12 months in both Pre-PC (−6.7 kg, −9.5 kg, –9.5 kg, and −8.6 kg at 3, 6, 9, and 12 months from 107.5 kg at baseline, *p*-values <0.001) and PC (−6.6 kg, −9.0 kg, −9.2 kg, and −8.3 kg at 3, 6, 9, and 12 months from 108.4 kg at baseline, *p*-values <0.001) patients (Table 2). Both cohorts showed a similar weight loss trajectory over time, as shown in Figure 2, with no significant differences (Table 2). This includes the early stages (approximately 120 days) of treatment which coexisted with the declaration of the pandemic and stay-at-home measures.

**Table 2:** Weight from baseline to 1 year in Pre-PC and PC.

	**Baseline**	**3 months**	**6 months**	**9 months**	**12 months**
Pre-PC	107.5 ± 0.5	100.8 ± 0.5***	98.0 ± 0.5***	98.0 ± 0.6***	98.9 ± 0.6***
PC	108.4 ± 0.5	101.8 ± 0.5***	99.4 ± 0.5***	99.2 ± 0.6***	100.1 ± 0.6***
Pre-PC vs. PC	−0.8 ± 0.6^ns^	−1.0 ± 0.7^ns^	−1.4 ± 0.8^ns^	−1.2 ± 0.8^ns^	−1.1 ± 0.8^ns^

*Pre-PC, Pre-pandemic cohort; PC, Pandemic cohort*.

Adjusted means and mean differences were obtained from linear mixed effects model (LMM) controlling for age, gender, baseline BMI, and HbA1c. A maximum likelihood-based approach was used to estimate missing data.*** p < 0.001 baseline vs. respective time points; ns not significant Pre-PC vs. PC.

There were no significant differences in percent weight loss at 1 year between the Pre-PC (7.9%) and the PC (7.5%) (*p* > 0.05) (*p* > 0.01, Supplementary Table 1), nor in the proportion of patients achieving weight loss of 5% or more between the Pre-PC (52.8% ≥ 5% loss) and the PC (55.5% ≥ 5% loss, *p* > 0.05, Supplementary Figure 1), although the proportion of individuals attaining weight loss of 10% or more was slightly greater in the Pre-PC than in the PC (Pre-PC, 32.2%; PC, 29.7%, *p* = 0.03, Supplementary Figure 1).

### Mobile Health App Use

To assess the differences in the mobile health app use between the Pre-PC and the PC, multiple indicators of daily app use from baseline to 1 year were compared between cohorts. As shown in Supplementary Table 1, there were significantly higher number of days with BHB loggings, and app usage from baseline to 1 year in PC versus Pre-PC. There was no difference in the number of daily weight loggings between the Pre-PC and the PC.

## Discussion

The present study assessed the effect of the COVID-19 pandemic on weight loss over 1 year in patients enrolled in a very-low-carbohydrate intervention delivered *via* telemedicine. Results demonstrated that the pandemic had no effect on the degree of weight loss, as there were no significant differences in the absolute or percent weight change between the Pre-PC and the PC at 1 year. Our findings support other studies that suggested that digitally supported programs were effective alternatives in mitigating some of the negative behavioral and lifestyle effects of government-enforced public health measures (15, 17). Likewise, telehealth can be considered a practical and efficacious healthcare delivery mode for chronic diseases during the COVID-19 pandemic (18).

Studies reported that the established or newly transitioned online weight loss programs were effective in short-term weight loss (15, 16). One behavioral weight loss intervention study changed the delivery mode from in-person to video conference during the lockdown, and reported that this change helped participants stay accountable and lose a significant amount of weight over the 5–6 weeks of the lockdown period, while only 8% of the participants reported a weight gain >1% (15). However, another program delivered online even prior to the COVID-19 pandemic reported a decline in weight loss in the first month of treatment among those who enrolled during the 7 weeks overlapping the onset of the pandemic and lockdown period (March 8 through 19 April 2020) when compared to those enrolled the year prior, although weight loss over 30 weeks was not impacted (16). This lag in weight loss, especially during the initial lockdown period, was not evident in our telemedicine intervention.

The weight loss trajectories of the Pre-PC and the PC were identical, except for the period between 150 and 250 days where the PC had a slightly less weight loss than the Pre-PC, although the difference in the degree of weight loss was not significant. This similar weight loss trend was maintained in the PC despite 15.7% of the patients reporting a COVID-19 diagnosis during the intervention period. We also found that the proportion of individuals attaining weight loss of ≥ 5% in the Pre-PC and the PC were not different, which is clinically meaningful to patients with T2D and prediabetes given that weight loss of 5% or more is associated with improvements in glycemic control and cardiovascular risk markers (21, 22). Considering the reported benefits associated with weight loss in reducing COVID-19 symptom severity including hospitalization (23–25), and since our intervention has been reported to improve obesity-related comorbidities like T2D and hypertension as well as markers of systemic inflammation (26, 27), future research should evaluate the relationship between weight loss induced by carbohydrate restriction and the severity of illness due to COVID-19 infection (28, 29).

Our study also revealed that the PC was more engaged in the mobile app through daily use and more logged BHB values throughout the 1-year treatment. It is plausible that the regular interaction through the health app provided the support patients needed during the stressful phase of the pandemic to stay focused on the intervention. We also speculate that the constant evolution of our platform and the nature of it enabling both proactive and reactive feedback tailored to patient need may have played a role in the increased engagement and aided in the maintenance of weight loss during the pandemic when other interventions were challenged. Several studies reported that increased social media interaction and smartphone app use during the pandemic, especially fitness app use, were associated with increase in and maintenance of physical activity (30, 31). Likewise, a recent study found that adoption of a remote consultation in an onsite weight loss intervention during the pandemic improved weight loss, and there was a dose-response effect between weight loss and the number of consultations (17).

This is the first study to our knowledge that assessed the impact of the COVID-19 pandemic and the lockdown period on the effectiveness of a well formulated NKI delivered *via* telemedicine for 1 year on weight loss. The follow-up length of 1 year allowed the lockdown and post-lockdown periods to be included in the assessment, where new waves of infection repeatedly challenged patients' social environment and behaviors. The use of nationwide, real-world data may aid generalizability for future waves and their associated public health measures. The main strength of this study was the use of propensity score matched groups based on baseline characteristics. While other studies utilized self-reported weight, this study utilized objective measurements of weight collected using a cellular connected scale that automatically uploaded values to the app. A limitation of this study is that the results may not be generalizable to a broader population of people with uncomplicated obesity, as it is possible that high prevalence of people with T2D who enroll in this intervention have different underlying characteristics and may be more motivated. It is also possible that some of the public health measures restricting dining outside of the home and engagement in social eating may have encouraged those who were motivated to enroll in the program during the pandemic.

## Conclusion

Despite the obesogenic environment observed during the pandemic, patients who enrolled in this intervention achieved clinically meaningful weight loss over 1 year that was no different from weight loss achieved by matched patients prior to the pandemic. Considering the continued presence of COVID-19 and its risk for people with T2D and obesity, remotely available and effective weight loss interventions may provide clinicians with an additional tool for weight control and COVID-19 risk management.

## Data Availability Statement

The datasets presented in this article are not readily available and reasonable request to access the data is considered if a proposal on how the data will be used is included in the request. Requests to access the datasets should be directed to shaminie@virtahealth.com.

## Ethics Statement

Ethics review and approval/written informed consent was not required since the data used for the analysis was de-identified and the study is not considered human subjects' research per local legislation and institutional requirements.

## Author Contributions

SA acquired and compiled the data, analyzed the data, and drafted the manuscript. RA drafted the manuscript. AM, MV, BV, SP, and RR edited the manuscript. All authors contributed to the article and approved the submitted version.

## Conflict of Interest

SA, RA, AM, MV, BV, SP, and RR were employed by Virta Health Corp and were offered stock options. SDP is one of the co-founders of Virta Health Corp.

## Publisher's Note

All claims expressed in this article are solely those of the authors and do not necessarily represent those of their affiliated organizations, or those of the publisher, the editors and the reviewers. Any product that may be evaluated in this article, or claim that may be made by its manufacturer, is not guaranteed or endorsed by the publisher.
