# Supplementary material for: Impact of the COVID-19 Pandemic on the Effectiveness of a Metabolic Health Telemedicine Intervention for Weight Loss: A Propensity Score Matching Analysis
Source: Front Public Health. 2022 Jun 16;10:897099. doi: 10.3389/fpubh.2022.897099 (PMC9246258; doi:10.3389/fpubh.2022.897099)

Impact of the COVID-19 pandemic on the effectiveness of a metabolic health telemedicine intervention for weight loss: A propensity score matching analysis

Shaminie J. Athinarayanan[1], Rebecca N. Adams[1], Michelle VanTieghem[1], Amy L McKenzie[1], Brittanie M. Volk[1], Robert E. Ratner[1], Stephen D. Phinney[1]

[1] Virta Health, 501 Folsom Street, San Francisco, CA 94105, USA

*** Correspondence:**
Shaminie J. Athinarayanan
shaminie@virtahealth.com

Supplementary Table 1. Percentage weight loss and app engagement variables from baseline to 1 year between the matched Pre-PC and PC

	**Pre-PC**	**PC**	**P-value**
**Percentage Weight Loss**			
Multiple Imputed	-7.9 ± 0.4	-7.5 ± 0.4	0.50
Completers with data	-8.0 ± 0.4	-7.5 ± 0.4	0.41
**App Use Variables**			
Weight Logging Days	272.0 (129.2)	288.8 (119.2)	0.05
BHB Logging Days	171.8 (79.6)	221.4 (103.6)	**<0.001**
App Use Days	245.6 (83.9)	263.8 (85.2)	**<0.01**

Abbreviations. Pre-PC, Pre-pandemic cohort; PC, Pandemic cohort; T2D, type 2 diabetes; BHB, beta-hydroxybutyrate; App, application. Note. Percent weight change was missing in 11.3% of the Pre-PC and 6.6% of the PC. Missing values were multiple imputed 40 times and a second analysis was performed in completers with data. Independent sample T-tests were used to compare means between Pre-PC and PC.

Supplementary Figure 1. Percentage of patients achieving weight loss thresholds in Pre-PC and PC

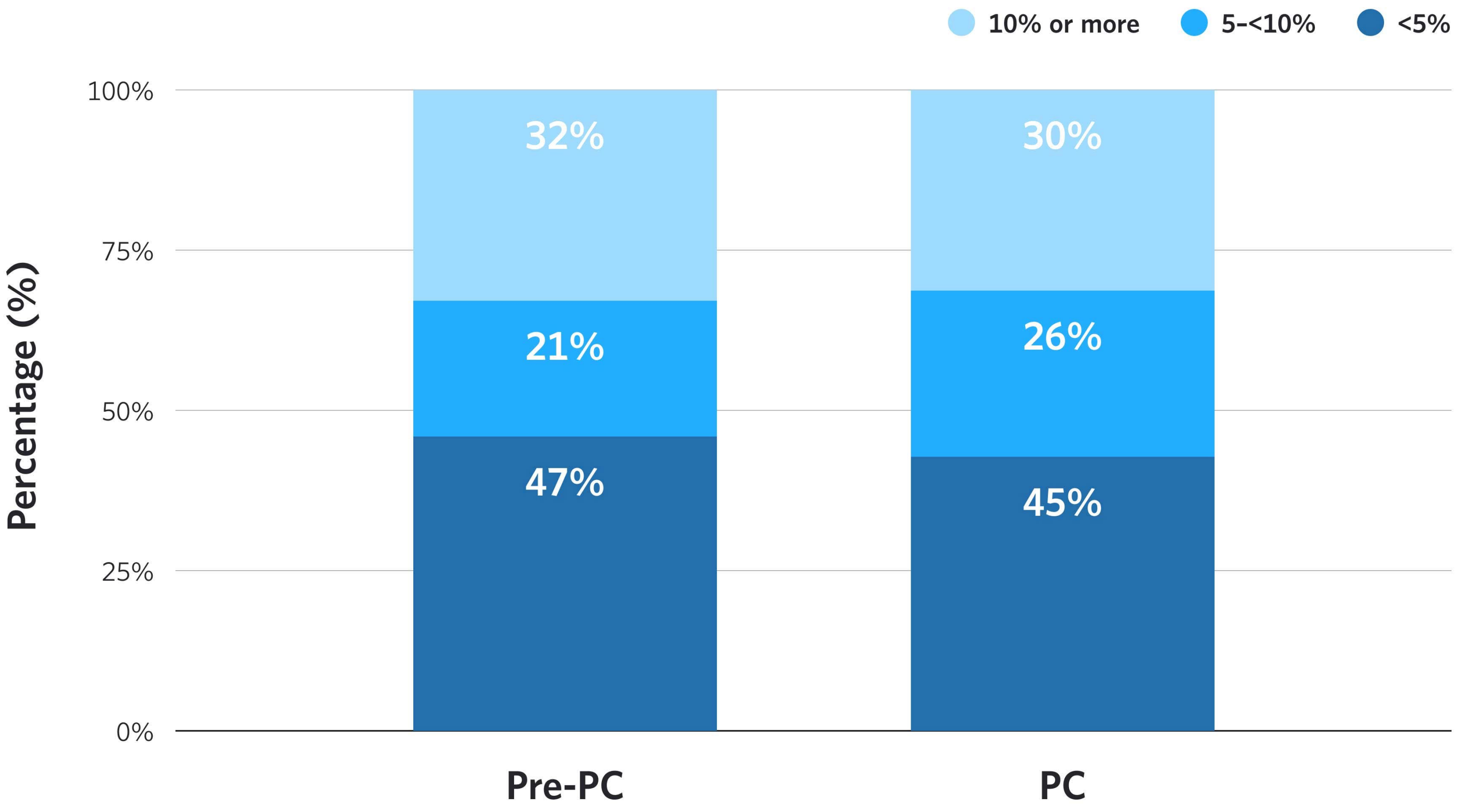

Supplement: Supplementary file 1 [file Data_Sheet_1.pdf]
